# The interdependence of isoprenoid synthesis and apicoplast biogenesis in malaria parasites

**DOI:** 10.1371/journal.ppat.1011713

**Published:** 2023-10-26

**Authors:** Megan Okada, Paul A. Sigala

**Affiliations:** Department of Biochemistry, University of Utah School of Medicine, Salt Lake City, Utah, United States; Children’s Hospital of Philadelphia, UNITED STATES

## Abstract

Isoprenoid precursor synthesis is an ancient and fundamental function of plastid organelles and a critical metabolic activity of the apicoplast in *Plasmodium* malaria parasites [1–3]. Over the past decade, our understanding of apicoplast properties and functions has increased enormously [4], due in large part to our ability to rescue blood-stage parasites from apicoplast-specific dysfunctions by supplementing cultures with isopentenyl pyrophosphate (IPP), a key output of this organelle [5,6]. In this Pearl, we explore the interdependence between isoprenoid metabolism and apicoplast biogenesis in *P*. *falciparum* and highlight critical future questions to answer.

## Cellular isoprenoid metabolism in *Plasmodium* parasites depends on the apicoplast

**Fig 1:**
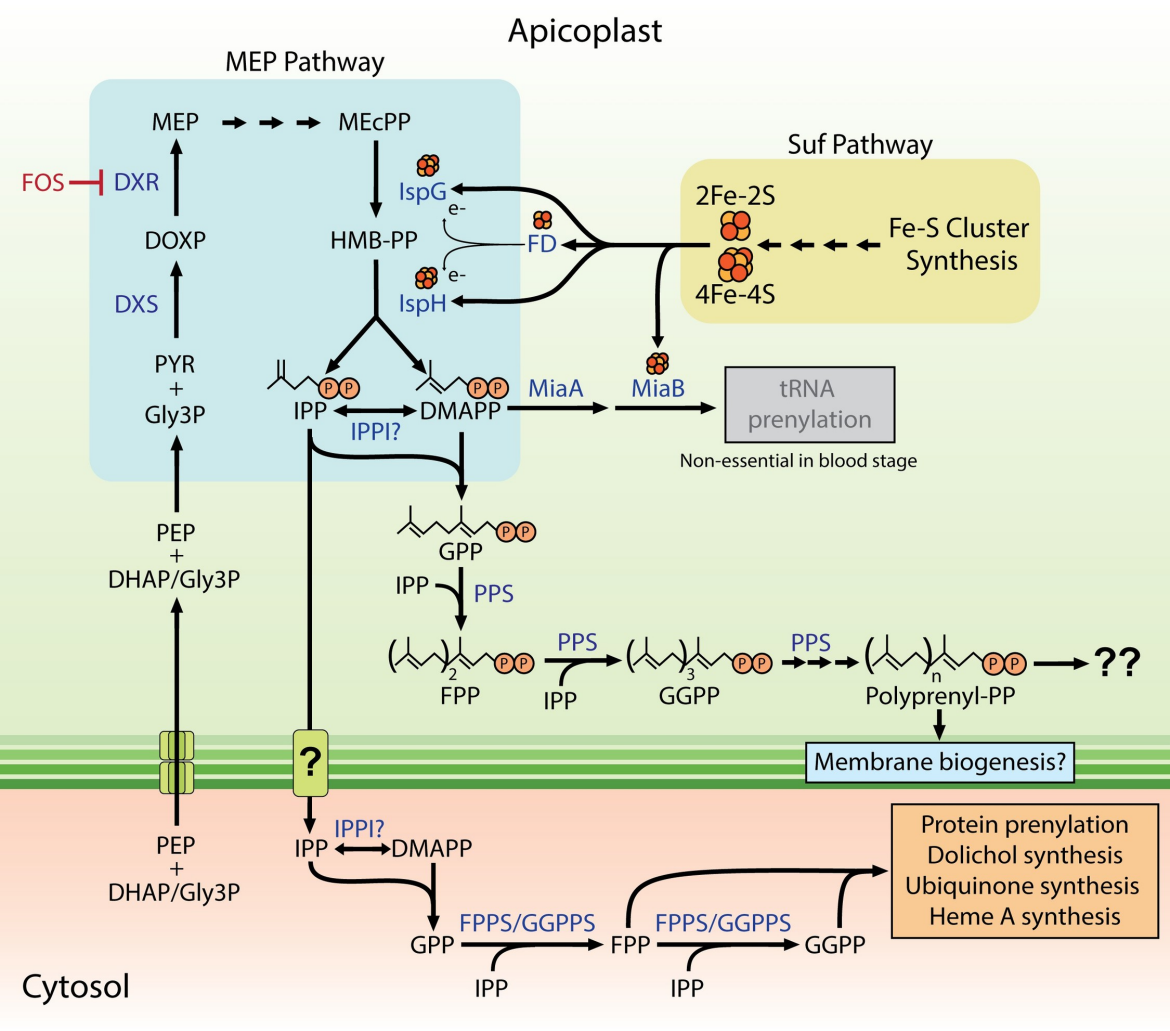
Isoprenoid synthesis and utilization by blood-stage malaria parasites. Abbreviations: DHAP, dihydroxyacetone phosphate; DMAPP, dimethylallyl pyrophosphate; DOXP, 1-deoxy-D-xylulose 5-phosphate; DXR, DOXP reductase; DXS, 1-deoxy-D-xylulose-5-phosphate synthase; FD, ferredoxin; FOS, fosmidomycin; FPP, farnesyl pyrophosphate; FPPS, FPP synthase; GPP, geranyl pyrophosphate; GGPP, geranylgeranyl pyrophosphate; GGPPS, GGPP synthase; Gly3P, glyceraldehyde-3-phosphate; HMB-PP, (E)-4-hydroxy-3-methyl-but-2-enyl pyrophosphate; IPP, isopentenyl pyrophosphate; IPPI, IPP isomerase; IspG, HMP-PP synthase; IspH, HMB-PP reductase; MEcPP, 2-C-methyl-D-erythritol-2,4-cyclopyrophosphate; MEP, methylerythritol phosphate; MiaA, tRNA dimethylallyltransferase; MiaB, tRNA 2-methylthio-N(6)-dimethylallyladenosine synthase; PEP, phosphoenolpyruvate; PPS, polyprenyl synthase; PYR, pyruvate. Question marks indicate uncertainty.

Malaria parasites retain a non-mevalonate/methylerythritol phosphate (MEP) pathway for isoprenoid precursor synthesis in the apicoplast organelle. This pathway synthesizes two 5-carbon isoprene subunits, isopentenyl pyrophosphate (IPP) and dimethylallyl pyrophosphate (DMAPP), whose sequential condensation is critical for making diverse longer-chain isoprenoids required for a variety of key cellular functions that include protein prenylation, dolichol synthesis for protein glycosylation, and biosynthesis of mitochondrial ubiquinone and heme A (Fig 1). Core enzymes in the MEP pathway are encoded in the nucleus but are trafficked to the apicoplast after translation in the parasite cytoplasm.

Within the apicoplast, isoprenoid precursor synthesis requires the support of several additional metabolic pathways. The final 2 enzymes in the MEP pathway, IspG and IspH, contain essential Fe-S cluster cofactors that receive electrons from apicoplast-targeted ferredoxin, which also contains an Fe-S cluster. These critical Fe-S cofactors are produced by the apicoplast Suf pathway, which is therefore essential for isoprenoid synthesis [7–10]. One of the Suf pathway proteins, SufB, is encoded on the apicoplast genome, and thus replication, transcription, and translation of the apicoplast genome are expected to be essential for Fe-S cluster provision to the MEP pathway. Substrates for isoprenoid precursor synthesis include glyceraldehyde-3-phosphate, pyruvate, and cytidine triphosphate, whose production and/or import into the apicoplast depend on apicoplast pyruvate kinase II and membrane transporters [11]. Because IPP and DMAPP synthesis requires the integrated function of MEP enzymes and supporting pathways within the apicoplast (Fig 1), disruption of the apicoplast organelle via biochemical and/or genetic perturbations ablates the synthesis of these key isoprenoid precursors [5,6]. Cellular isoprenoid synthesis in *Plasmodium* therefore requires the apicoplast and the pathways that support and maintain this key organelle.

## Apicoplast biogenesis requires isoprenoid precursor synthesis and the pathways that support MEP pathway activity

Prior studies in *Plasmodium* focused almost exclusively on cellular roles for isoprenoids outside of the apicoplast, since exogenous IPP can rescue parasites from loss of the apicoplast and the attendant ablation of endogenous IPP synthesis. Nevertheless, multiple prior papers reported that MEP pathway inhibitors like fosmidomycin (FOS) blocked apicoplast biogenesis and prevented elongation of the organelle in blood-stage parasites [12–14]. Recent work demonstrated that these defects were due to specific apicoplast dysfunction (rather than a nonspecific phenotype of dying parasites) and confirmed a critical role for isoprenoids in supporting apicoplast biogenesis [15]. The only previously annotated role for isoprenoids in the apicoplast was tRNA prenylation by MiaA, but knockout of this enzyme had no impact on apicoplast biogenesis or parasite viability (Fig 1). However, it was discovered that *Plasmodium* parasites target a polyprenyl synthase (PPS) enzyme to the apicoplast that synthesizes longer-chain linear isoprenoids required for organelle biogenesis [15]. Indeed, parasites could be rescued from loss of PPS by addition of long-chain decaprenol (C_50_-OH) but not by GGOH (C_20_-OH) or shorter polyprenols.

It remains a key unmet challenge to understand why apicoplast biogenesis requires long-chain polyprenols. These compounds can serve multiple functions in plant chloroplasts (e.g., light harvesting and photosynthesis, oxidative stress protection, signaling and defense molecules), but there is little or no evidence for these functions in *Plasmodium* parasites [1,3,15]. In the absence of other known roles for long-chain linear isoprenoids, one hypothesis is that these lipids tune the structure and flexibility of apicoplast membranes, which is a known or proposed biophysical role for membrane polyprenols in bacteria, plants, and other eukaryotes [16]. The evolutionary pathway by which the *Plasmodium* apicoplast acquired and retained a PPS enzyme is also unclear. Long-chain isoprenoids like undecaprenyl phosphate serve as the membrane lipid carrier for bacterial peptidoglycans used in cell wall synthesis [16,17]. This pathway (which includes a PPS) was likely present in the ancestral prokaryotic progenitor of the apicoplast, with most enzymatic components being lost during evolution of the current organelle. We hypothesize that PPS may have been retained in the *Plasmodium* apicoplast as a vestige of this erstwhile peptidoglycan synthesis pathway, due to a separate essential role for long-chain polyprenols in tuning membrane biogenesis.

Due to its reciprocal dependence on isoprenoid precursor synthesis, apicoplast biogenesis also requires organelle functions that support MEP pathway activity, including Suf-mediated Fe-S cluster biogenesis, replication of the apicoplast genome and expression of SufB, pyruvate kinase II activity, and membrane transporters that import the requisite biosynthetic substrates (Fig 1) [4]. Several of these pathways also have separate essential roles in apicoplast biogenesis in addition to and independent of their direct support of MEP pathway activity. In the next section, we explore some of these pathways and how parasite rescue by exogenous IPP has been used to test and understand the role of apicoplast-targeted proteins in organelle biogenesis.

## IPP rescue as a tool to probe apicoplast-specific function and organelle biogenesis

**Fig 2:**
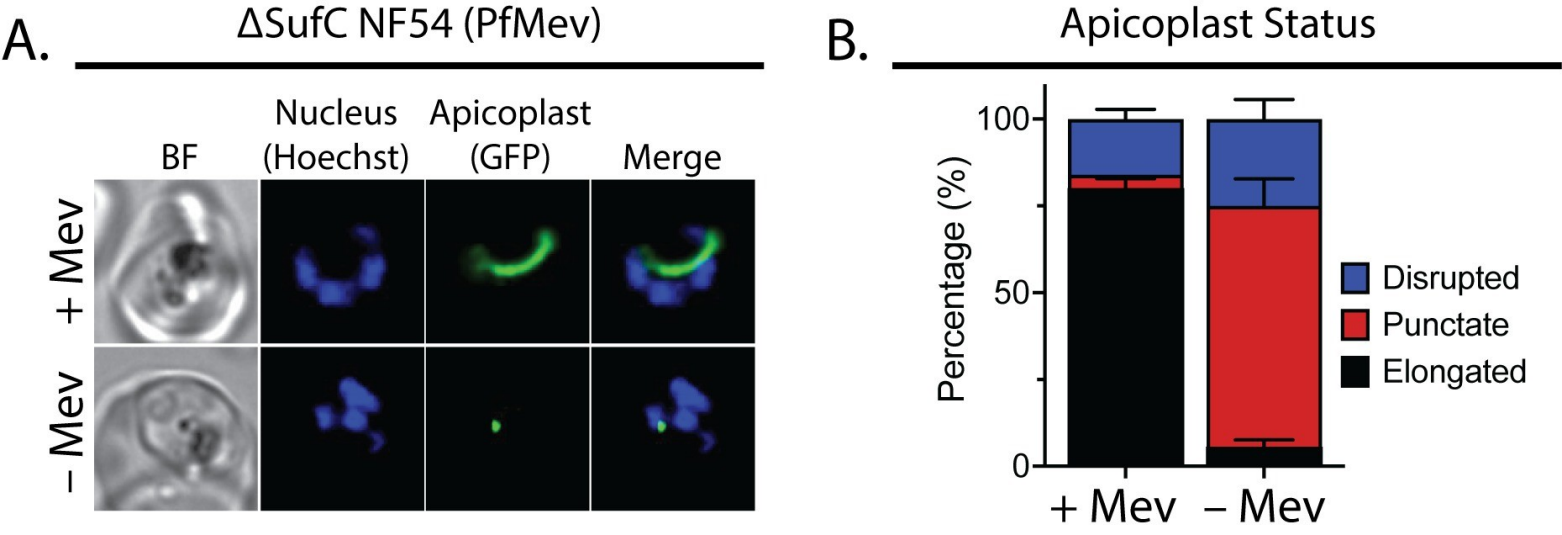
Apicoplast Fe-S cluster biosynthesis is required for isoprenoid synthesis and apicoplast biogenesis. (**A**) Live-cell imaging of ∆SufC NF54 (PfMev) parasites synchronized and cultured 36 hours with or without 50 μM DL-mevalonate (Mev) to stimulate IPP synthesis [7]. (**B**) Population analysis of parasites imaged under the conditions in (**A**). A total of 56 parasites were imaged per condition from biological duplicate samples. Apicoplast morphologies were scored as punctate, elongated, or disrupted (dispersed signal) and plotted by population percentage.

The foundational discovery that exogenous IPP could rescue parasites from apicoplast-specific dysfunctions and organelle loss enabled many opportunities to probe the functional properties of the apicoplast and its constituent proteins [5]. Because the apicoplast requires isoprenoid precursors to initiate and complete its biogenesis program [15], 2 general phenotypes have been observed when parasites are rescued by exogenous IPP from chemical or genetic perturbations to the apicoplast. For proteins whose only essential function in blood-stage parasites is to support isoprenoid precursor biosynthesis, including MEP-pathway enzymes (e.g., DXS) and several proteins in the Suf pathway (e.g., SufC) [7,11], IPP bypasses the loss or inhibition of these proteins to rescue parasites and support normal apicoplast elongation and division. In such cases (e.g., FOS treatment, ∆DXS, ∆SufC), however, defective apicoplast biogenesis is observed when IPP supplementation is withheld such that the punctate apicoplast present in rings and trophozoites fails to elongate as parasites progress into schizogeny (Fig 2) [7,15]. For apicoplast-targeted proteins that have essential functions in apicoplast biogenesis that are in addition to or apart from supporting MEP-pathway activity (e.g., ClpC, PPS, SufS), IPP rescues parasite viability from loss of these proteins but the apicoplast fails to elongate and divide such that daughter parasites stably lose the organelle and its genome [7,15,18].

Current knowledge specifies IPP and coenzyme A (CoA) as the only biosynthetic outputs of the apicoplast that are essential for blood-stage *P*. *falciparum* [5,19,20]. Unlike IPP synthesis, however, CoA biosynthesis does not require the apicoplast organelle as a metabolic hub and appears to persist regardless of apicoplast maintenance or loss or targeting of the terminal enzyme to the apicoplast, cytoplasm, or vesicles [19]. Nevertheless, apicoplast biogenesis appears to depend on synthesis of both IPP and CoA [15,19]. Based on these observations, we propose that any essential apicoplast-targeted protein in blood-stage *Plasmodium* will be required for organelle maintenance due to a critical role in IPP synthesis and/or in other aspects of apicoplast biogenesis apart from IPP synthesis. Future studies of apicoplast functions can test this prediction and extend or revise its validity to different parasite stages in distinct host environments, where additional apicoplast pathways like type II fatty acid synthesis can produce essential outputs [21,22] that may also be required for apicoplast biogenesis.

### Conservation and divergence in apicomplexan isoprenoid metabolism

The MEP pathway for isoprenoid precursor synthesis is a hallmark of apicoplast retention in apicomplexan parasites [1]. Although isoprenoid synthesis and apicoplast biogenesis are reciprocally coupled in *Plasmodium*, this paradigm of metabolic interdependence does not appear to hold throughout the phylum Apicomplexa. *Toxoplasma gondii* parasites express 2 PPS homologs but neither appears to localize to the apicoplast [15,23,24]. Although the MEP pathway is essential for *T*. *gondii*, loss of isoprenoid precursor synthesis has no apparent impact on apicoplast biogenesis [12,25]. These metabolic and phenotypic differences between *Plasmodium* and *Toxoplasma* presumably reflect the differing selective pressures that guided evolution of these parasites over the 600 Myr timeline of their divergence from a common ancestor. Multiple PPS homologs are also retained by other hematozoan parasites (e.g., *Babesia*) that are more closely related to *Plasmodium* than *Toxoplasma*, but a possible role for PPS in apicoplast biogenesis is unexplored in these organisms. Understanding the similarities and differences in isoprenoid metabolism between broader apicomplexan parasites can unveil and unravel the biochemical forces that have spurred evolution of intracellular parasitism along distinct pathways.

### Frontier questions

Below, we lay out several key questions at the frontier of our understanding of the apicoplast and isoprenoid metabolism in *P*. *falciparum* parasites that we hope will stimulate future studies in these areas.

1. *Why does apicoplast biogenesis require long-chain linear isoprenoids*? We hypothesize that these lipids are critical to tune the biophysical properties and flexibility of apicoplast membranes, such that their deficiency impairs membrane expansion during organelle biogenesis. It remains unknown if these isoprenoids are enriched in specific or all apicoplast membranes and/or serve other roles in organelle biogenesis. It may be possible to test our hypothesis with small-molecule dyes whose fluorescence properties are sensitive to variations in membrane fluidity, together with chemical rescue experiments using a homologous series of progressively longer-chain polyprenols to bypass loss of PPS.
2. *How are isoprenoids transported into and out of the apicoplast*? Isoprenoid precursors synthesized in the apicoplast, including IPP and DMAPP, are required and utilized outside the organelle. Chemical rescue experiments suggest that IPP and longer isoprenoids can enter the apicoplast. Charged metabolites face an unfavorable energetic barrier to diffuse across the 4 hydrophobic membrane bilayers that encircle the apicoplast, but specific protein transporters that enable their passage into and out of the organelle remain undefined.
3. *What prenyl synthase supports the biosynthesis of ubiquinone required in the Plasmodium mitochondrion*? Ubiquinone, which has a polyprenyl tail, is critical for function of the mitochondrial electron transport chain. A mitochondrial-targeted polyprenyl synthase has been identified in *T*. *gondii* [23], but the metabolic origins of this critical coenzyme and its polyprenyl tail in *Plasmodium* remain obscure and poorly studied. Apicoplast PPS was proposed to play this role in *Plasmodium* [26], but the ability of IPP to rescue parasites from PPS knockdown argues against such a role [15].
4. *Are there additional metabolic uses of isoprenoids by malaria parasites beyond currently defined products*? *Plasmodium* lacks enzyme homologs for terpene and carotenoid biosynthesis, and recent work found no evidence to support IPP incorporation into carotenoids [2,3,15]. Nevertheless, are there other metabolic end products in parasites that depend on IPP synthesis and remain to be discovered?
